# *Lactobacillus rhamnosus* JY02 Ameliorates Sarcopenia by Anti-Atrophic Effects in a Dexamethasone-Induced Cellular and Murine Model

**DOI:** 10.4014/jmb.2303.03001

**Published:** 2023-03-27

**Authors:** Juyeon Lee, Minkyoung Kang, Jiseon Yoo, Sujeong Lee, Minji Kang, Bohyun Yun, Jong Nam Kim, Hyoungsun Moon, Yihyung Chung, Sangnam Oh

**Affiliations:** 1 Department of Functional Food and Biotechnology, Jeonju University, Jeonju 55069, Republic of Korea; 2 Division of Practical Application, Honam National Institute of Biological Resources, Mokpo 58762, Republic of Korea; 3 Department of Food Science and Nutrition, Dongseo University, Busan 47011, Republic of Korea; 4 EN Food Contents Inc., Gimje 54379, Republic of Korea; 5 Jeonbuk Institute for Food-Bioindustry, Jeonju 54810, Republic of Korea

**Keywords:** Probiotics, sarcopenia, muscle atrophy, *Lactobacillus rhamnosus*, dexamethasone

## Abstract

Sarcopenia is defined as loss of muscle mass and strength due to aging. Recent studies show that sarcopenia may improve via the gut–muscle axis, suggesting that gut health may affect muscle phenotypes. In this study, we aimed to investigate the ability of *Lactobacillus rhamnosus* JY02 as a probiotic strain isolated from kimchi to alleviate sarcopenia. *L. rhamnosus* JY02-conditioned medium (CM) reduced dexamethasone (DEX)-induced myotube diameter atrophy and expression of muscle degradation markers (MuRF1 and atrogin-1) in C2C12 cells. The amelioration of sarcopenia was investigated by measuring body composition (lean mass), hand grip strength, myofibril size (using histological analysis), and mRNA and protein expression of muscle-related factors in a DEX-induced mouse model. The results of these analyses showed that *L. rhamnosus* JY02 supplementation promoted the production of muscle-enhancement markers (MHC Iβ, MHC IIα, and Myo-D) and reduced both the production of muscle degradation markers and the symptoms of muscle atrophy (loss of lean mass and muscle strength). We also found decreased levels of pro-inflammatory cytokines (IL-6, IFN-γ) and increased levels of anti-inflammatory cytokines (IL-10) in the serum of DEX+JY02-administered mice compared to those in DEX-treated mice. Overall, these results suggest that *L. rhamnosus* JY02 is a potent probiotic supplement that prevents sarcopenia by suppressing muscle atrophy.

## Introduction

Muscle atrophy, defined as age-related loss of muscle mass and function, is frequently observed in sarcopenia, a syndrome characterized by progressive and adverse muscle changes [1]. Maintaining muscle functiofn is essential for healthy aging, as muscular atrophy may increase the risk of fractures, falls, and other complications [2-5]. While there are currently no therapeutics that have shown efficacy in treating sarcopenia, factors such as nutrient absorption, energy metabolism, immunity, and insulin sensitivity through intestinal microbes have been found to have a direct or indirect effect on muscle phenotype [6-8]. In particular, as decreased gut function is associated with skeletal muscle atrophy, targeting this by consuming probiotics may help alleviate sarcopenia [9].

Probiotics are viable microorganisms that reach the intestines in an active state in adequate amounts and provide health benefits to the host [10]. *Lactobacillus*, the most studied probiotic strain, has anti-inflammatory, anticancer, and antioxidant effects. In addition, it helps with immune enhancement, oxidative stress reduction, and improvement of intestinal barrier function [7, 11-17]. Probiotics regulate several diseases by modulating gut microbiome composition and producing metabolites, including branched-chain amino acids (BCAAs) and short-chain fatty acids (SCFAs) [11, 18, 19]. Additionally, supplementation with *Lactobacillus* spp. has been shown to enhance the expression of markers inducing myoblast differentiation and significantly lower levels of ubiquitin-proteasome E3 ligases, such as atrogin1 and MuRF1 and the specific cytokine TNF-α. This has been confirmed to cause skeletal muscle loss [20-23].

Dexamethasone (DEX) is a synthetic glucocorticoid used to treat autoimmune diseases such as inflammation, allergies, and arthritis [24, 25]. However, prolonged exposure therapy to high doses may cause skeletal muscle atrophy [25, 26]. DEX induces enhancement of the mRNA expression of muscle RING-finger protein-1(MuRF1) and atrogin-1 genes, as well as muscle-specific E3 ubiquitin ligases involved in the ubiquitin-proteasome system, and decreases the diameter of C2C12 myotubes [27, 28]. Moreover, an increase in reactive oxygen species (ROS) by DEX treatment promotes the ubiquitination of muscle proteins by increasing the expression of E3 ligase and muscle atrophy caused by mitochondrial dysfunction [29]. Consequently, several studies have used dexamethasone to induce muscle atrophy both in vitro and *in vivo* [30-32]. Accordingly, we also evaluated the muscle improvement effect of *L. rhamnosus* JY02 in C2C12 muscle cells and C57BL/6 mice using DEX as a muscle atrophy inducer.

Based on the gut–muscle axis, several studies have reported that probiotics induce positive changes in gut microbiome composition, suggesting a link between gut health and muscle homeostasis [33, 34]. Intestinal bacteria or microbiota composition may affect muscle protein synthesis, mitochondrial biogenesis, ROS production, inflammation, and muscle glycogen storage [34]. Chen *et al*. revealed that *Lactobacillus casei* Shirota supplementation had anti-sarcopenic effects by increasing the levels of several SCFAs and IL-10 and decreasing the levels of ROS, TNF-α, and pro-inflammatory cytokines via gut–muscle mediation [35]. SCFAs (such as acetates and butyrates) produced by probiotics act as energy substrates for colonic and intestinal epithelial cells and regulate the intestinal barrier function [36]. Thus, the gut microbiome regulates systemic inflammation, immunity, and energy metabolism, whereas aging-induced changes in the gut microbiome can affect host muscle mass and function [37]. In this study, we investigated the possibility that probiotic strains isolated from kimchi could induce muscle growth by improving intestinal health.

## Materials and Methods

### Cell Culture and Differentiation

Mouse myoblast C2C12 skeletal muscle cells were cultured in high-glucose Dulbecco’s modified Eagle’s medium (DMEM; WelGENE Inc., Korea) supplemented with 10% fetal bovine serum (FBS) and 1% Antibiotic-Antimycotic (Gibco, Paisley, Scotland) contains 10,000 units/ml of penicillin, 10,000 μg/ml of streptomycin, and 25 μg/ml of Gibco Amphotericin B. The cells were maintained at 37°C in a humidified atmosphere containing 5%CO_2_. C2C12 myotubes were induced from C2C12 myoblasts using a differentiation medium (DM) composed of high-glucose DMEM supplemented with 2% horse serum (Sigma-Aldrich, USA) and 1% antibiotics. When the myoblast density reached 90% confluence, the growth medium was changed to DM. All differentiation experiments were performed for 6 days, and the DM was changed every 2 days.

### Manufacture of Conditioned Media Using *L. rhamnosus* JY02

Cultured *L. rhamnosus* JY02 cells were centrifuged and washed with phosphate-buffered saline (PBS). After removing the supernatant, an equal amount of DMEM as compared to the culture medium was added to the remaining pellet, which was incubated at 37°C for 24 h. After 24 h, the supernatant was harvested by centrifugation (3,500 ×*g* for 10 min at 25°C). The pH of the obtained supernatant was adjusted to 7 using a pH meter for the cell experiments. The cell-free supernatant of DMEM medium obtained following bacterial culture. Conditioned media (CM) adjusted to pH 7 was used in subsequent experiments after sterilization using a 0.22 μm pore size syringe filter.

### MTT Assay

Cells were seeded at 1 × 10^4^ cells/cm^2^ in 24-well plates for 24 h. Cells were then incubated in a medium containing various concentrations of CM for 24 h. Then, 0.5 mg/ml of MTT reagent (M5655-500MG, Sigma) was added to each well for 2 h. After the cell medium was removed, formazan was dissolved in 1 ml dimethyl sulfoxide (DMSO). Next, 100 μl of the supernatant was aliquoted into a 96-well plate. The absorbance was measured at 570 nm. Cell viability was calculated as the ratio of the sample absorbance to the control absorbance.

For the myotube MTT assay, the cells were seeded at 4 × 10^4^ cells/cm^2^ in 24-well plates and incubated until a monolayer was formed. The medium was changed to DM for 6 days. The myotubes were treated with various concentrations (0.01, 0.1, 1, 2, 4, 8, or 10%) of CM for 24 h with 100 μM of Dexamethasone (DEX; Sigma-Aldrich, USA). After 24 h, an MTT assay was performed, and cell viability was measured.

### Giemsa Staining

Cells were seeded in 6-well plates (4 × 10^4^ cells/cm^2^) and incubated in growth medium until they reached 90%confluency. For visualization of myotubes and nuclei, the myotubes were stained with Giemsa dye. The myotubes were washed with cold PBS and fixed in 4% paraformaldehyde for 10 min. The cells were then washed twice with cold phosphate-buffered saline (PBS). For Giemsa staining, myotubes were incubated with 1 ml of modified Giemsa staining solution (51811-82-6, Sigma) diluted 1:20 in distilled water for 40 min at room temperature (RT). After 40 min, the myotubes were washed three times with distilled water. The myotubes and nuclei were then observed using an Olympus IX53 microscope. The average myotube diameter was calculated as the median value of the measured values using (Image J 1.48V – Java 1.6.0_20(32-bit), National Institute of Health, USA).

### Immunofluorescence and Fusion Index Determination

For immunostaining, differentiated C2C12 myotubes were fixed with 4% paraformaldehyde and incubated for 20 min at RT. The myotubes were then washed twice with PBS and permeabilized with 0.25% Triton X-100 for 5 min at RT. Following this, the myotubes were washed once with PBS and treated with 1% bovine serum albumin for 45 min at RT. The sections were then incubated with anti-myosin heavy chain primary antibodies (cat. No. sc-376157; 1:200) for 1 h at RT. After washing three times for 5 min each with PBS, diluted Alexa Fluor 488-conjugated (cat. No. 4408S; 1:1,000) secondary antibodies were added to each well and incubated at RT for 1 h in the dark. These were again washed three times with PBS for 5 min, after which diluted DAPI solution for nuclei staining was added to each well for 5 min at RT. Subsequently, the mounting medium was dropped onto a plate and covered with a cover glass. Images of the C2C12 myotubes were obtained using a fluorescence microscope (IX53; Olympus, Japan).

### Total RNA Isolation and Quantitative Reverse Transcription PCR (RT-qPCR)

Myotubes were harvested using the QIAzol Lysis Reagent (Qiagen, Germany). Total RNA was extracted using the AccuPrep Universal RNA Extraction Kit (Bioneer, Korea) according to the manufacturer’s instructions. One microgram of total RNA was used to synthesize cDNA using the iScript cDNA Synthesis Kit (Bio-Rad, USA), following the manufacturer’s protocols. RT-qPCR was performed on a StepOnePlus Real-Time PCR System using the Luna Universal qPCR Master Mix (New England Biolabs, USA). The gene expression level was normalized to that of β-actin, a housekeeping gene, using the ΔΔCt method.

### Western Blotting Analysis

Myotubes were lysed using a protein lysis buffer, and protein concentrations were quantified using the Bradford assay. Subsequently, heat-denatured cell lysates were subjected to sodium dodecyl sulfate (SDS) polyacrylamide gel electrophoresis (PAGE) and western blot analyses. Equal quantities (30 μg) of protein were loaded into each lane and subjected to SDS-PAGE on a 12% polyacrylamide gel. After electrophoresis, the proteins were transferred to polyvinylidene difluoride (PVDF) membranes and blocked with TBST buffer containing 5% skim milk overnight at 4°C. Membranes were incubated with the following primary antibodies: MuRF1 (cat. No. sc-398608; 1:250) at 4°C overnight. After 3 washes of 20 min each with TBST buffer, the membranes were incubated with the corresponding fluorescently labelled secondary antibodies (cat. No. sc-525409; 1:5000) at room temperature for 2 h. After washing, the protein bands were visualized using an ECL Western Blotting Detection system (Azure Biosystems 280, Inc., USA).

### Animal Experiments

C57BL/6 mice (7-week-old, male) were purchased from SLC (Japan). Mice were acclimatized and maintained under the following conditions: relative humidity, 55%; temperature, 23 ± 3°C; and a 12 h light/dark cycle. Six mice were randomly assigned and housed in standard laboratory cages, with free access to food and water. Mice were adapted for 7 days and then administered saline (Normal and DEX groups) or JY 02 suspension (1 × 10^8^ CFU/mouse, 0.1 ml/mouse; JY02 + DEX group) via oral gavage daily for 5 weeks. From day 28 to day 37, DEX (20 mg/kg) was intraperitoneally injected once per day into the mice in the DEX or JY02 + DEX groups. On day 37, the mice were sacrificed, and three muscle tissues, quadriceps (QD), gastrocnemius (GC), and tibialis anterior (TA), were harvested.

All of the animal experiments were carried out under the approval of the Animal Ethics Committee of Jeonju University and the animal protocols in this study were approved by the Institutional Animal Care and Use Committee of The Food Industry Promotional Agency of Korea (Approval number: IACUC-22-004).

### Assessment of Body Composition and Grip Strength

Lean body mass analysis was performed once every 2 weeks using nuclear magnetic resonance (Echo MRI) NMR (EchoMRI-700, Echo-MRI, USA). Grip strength was measured by placing the mouse on a grid connected to a grip strength meter (cat. No. BIO-GS3; Bioseb, France) and pulling the tail while holding the grid. Three measurements were performed, and the average value was obtained for each trial.

### Histological Analysis

To assess muscle tissue damage, the harvested tissues were fixed with 4% formaldehyde and then embedded in paraffin for histological assessment. Sections of the QD, GC, and TA were stained with hematoxylin and eosin (H&E). After staining, the cross-sectional area of the muscle fiber was quantified.

### Cytokine/Chemokine Analysis Using Cytometric Bead Array (CBA)

The concentrations of TNF-α, IL-6, IL-10, IL-12p70, IFN- γ, and MCP1 in serum were determined using a Cytometric Bead Array system (Mouse Inflammation Kit, CBA; BD Biosciences) according to the manufacturer’s instruction. Briefly, 50 μl of mixed capture beads and 50 μl of PE detection reagent were added for each sample. Then, the assay tube was incubated for 2 h in a dark room at RT. The assay tube was washed with 1 ml of wash buffer and centrifuged at 200 ×*g* for 5 min. Finally, the supernatant was discarded, and the bead pellet was resuspended in 0.3 ml of wash buffer. The sample was analyzed on a Beckman Coulter CytoFLEX Flow Cytometer using the supplied cytometer setup beads and the cytoExpert software.

### Statistical Analysis

Data are expressed as the mean ± standard deviation (SD). Statistical analyses were performed using one-way analysis of variance (ANOVA), followed by Dunnett’s test and Tukey’s multiple comparison tests using GraphPad Prism software (USA version 6.07). Differences compared with the control were considered statistically significant at *p* < 0.01.

## Results and Discussion

### *L. rhamnosus* JY02 Ameliorates C2C12 Myotube Atrophy.

*L. rhamnosus* JY02, which was isolated from kimchi, has probiotic properties and shows acid tolerance, bile tolerance, and intestinal adhesion (Fig. S1). We observed that JY02 prolonged lifespan (Fig. S2) and improved muscle function using *Caenorhabditis elegans* in an in vivo model (Fig. S3). Based on previous reports, probiotics produce surface molecules and metabolites that regulate and improve barrier function in intestinal epithelial cells; furthermore, it has been suggested that probiotics-derived molecules affect muscle health [4, 38, 39].

**Fig. 1:**
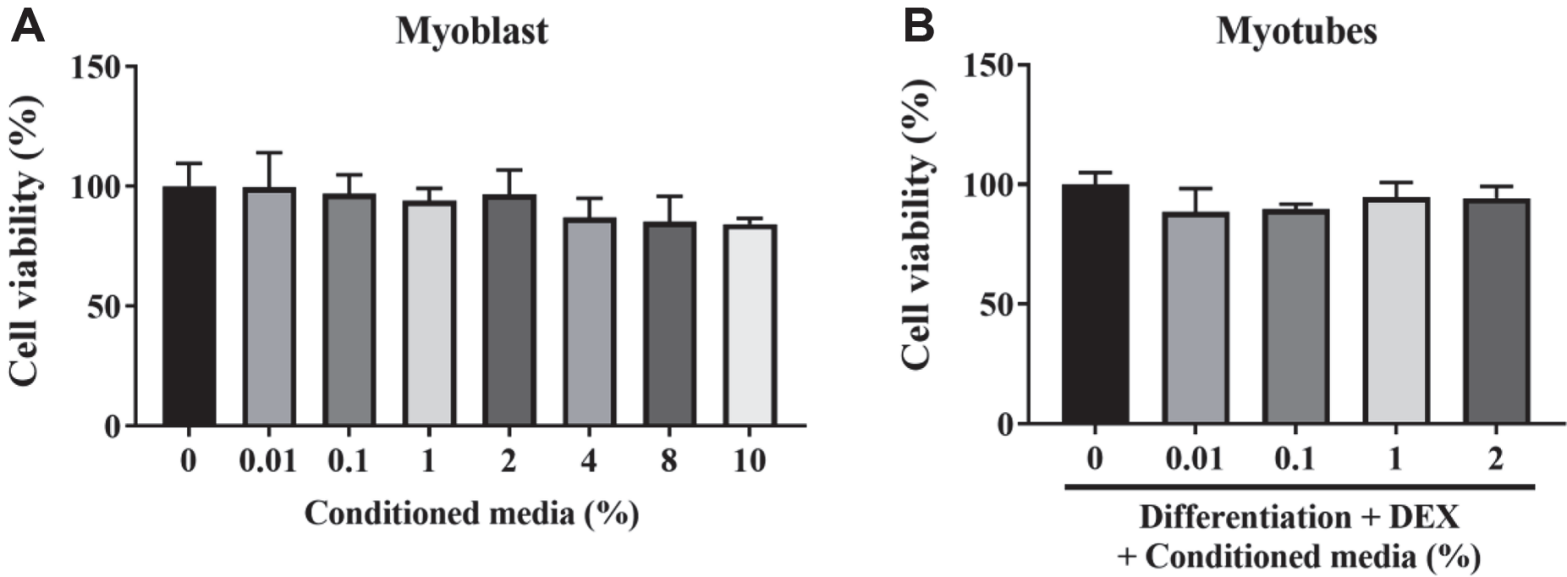
Measurement of cell viability. The viability of C2C12 myoblasts and myotubes after CM treatment with *L. rhamnosus* JY02. (**A**) MTT assay results obtained after 24 h of the incubation of C2C12 myoblasts with 0.01%–10% CM. (**B**) MTT assay results obtained after 24 h of the coincubation of C2C12-differentiated myotubes with CM and 100 μM DEX.

To investigate whether *L. rhamnosus* JY02 has an atrophying effect on myocytes at a cellular level, we prepared a conditioned medium (CM) of *L. rhamnosus* JY02 and treated C2C12 cells with various concentrations of the CM. The viability of myoblasts and myotubes was assessed to determine the appropriate treatment concentration of the JY02-CM. Myoblasts and myotubes treated with 0.01–2% CM maintained a high survival rate (>97%) compared with that of the untreated control group and the subsequent experiments were performed using this concentration (Figs. 1A and 1B).

**Fig. 2:**
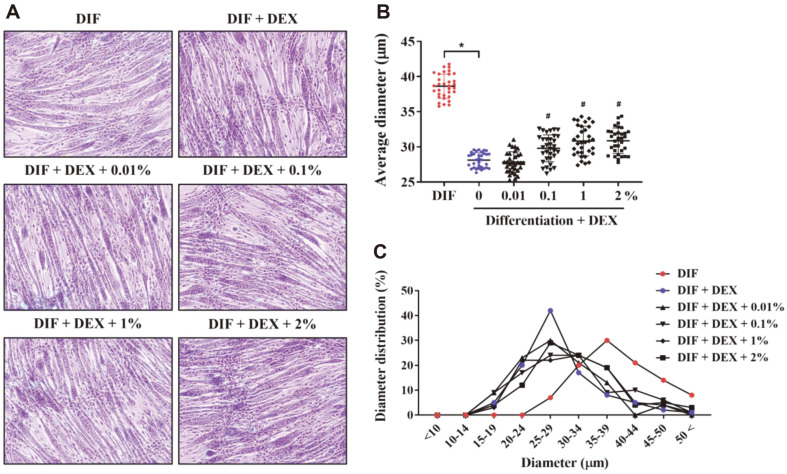
Myotube visualization using Giemsa staining. (**A**) Evaluation of myotube diameter by Giemsa staining after cotreatment of C2C12 myotubes with CM and 100 μM DEX for 24 h. (**B, C**) Mean myotube widths and myotube diameter distribution are presented. Myotube width was measured using ImageJ software; 100 myotubes were measured for each concentration and the median value was calculated as a representative value. Data are expressed as the mean ± SD. *p < 0.01 vs. DIF control, #*p* < 0.01 vs. 0%.

To determine whether treatment with JY02-CM alleviates DEX-induced myotube atrophy, C2C12 myotubes were visualized by Giemsa staining, which revealed that myotube diameter was significantly decreased by DEX treatment compared with that of the DIF control without DEX treatment. In contrast, the diameter of the canal atrophied by DEX significantly increased in the samples treated with 0.1, 1, or 2% CM (Figs. 2A-2C). These results suggest that the bioactive compound derived from *L. rhamnosus* JY02 can prevent DEX-induced atrophy in C2C12 myotubes. The possible active substance as a molecule that inhibits cellular muscle atrophy is estimated to be short-chain fatty acids, bacteriocins, vitamins, amino acids, oligosaccharides, and exopolysaccharides [40].

### *L. rhamnosus* JY02 Induces C2C12 Myogenic Differentiation and Suppresses the Expression of Muscle-Specific Ubiquitin Ligases in C2C12 Myotubes

**Fig. 3:**
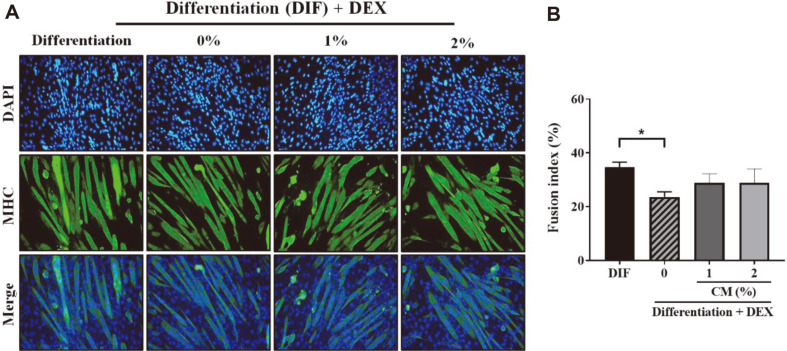
Immunofluorescence and fusion index determination. (**A**) Immunofluorescence staining of MHC (green) and DAPI (blue) in C2C12 myotubes. C2C12 cells were differentiated for 6 days and co-treated with DEX and CM for 24 h. Fusion index (%) = (number of nuclei in myotubes) / (total number of nuclei in myoblasts and myotubes) × 100.

Myosin heavy chain (MHC) is an essential factor involved in muscle contraction, and loss of skeletal muscle mass occurs when the shape of the MHC is altered or its size is reduced [41, 42]. MHC is a significant protein constituting myotubes, and a decrease in MHC levels indicates muscle atrophy [43]. To confirm the hypertrophic effect of CM on C2C12 myotubes, we stained the tissue samples for MHC [44]. We also measured the fusion index by immunofluorescence staining of the nucleus and MHC to confirm the expression level of MHC. Immunofluorescence assay with MHC antibody revealed the presence of MHC and the fusion of C2C12 myotubes containing multiple nuclei (Fig. 3A). The fusion index for myotubes treated with 100 μM DEX and 1% or 2% CM increased compared with that of the control group treated with only DEX. This result confirmed that JY02-CM promoted the differentiation of muscle cells.

**Fig. 4:**
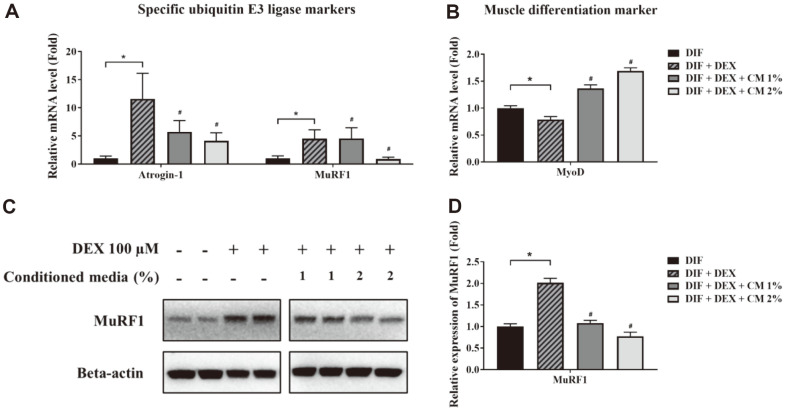
mRNA and protein expression levels of muscle markers in C2C12 myotubes. (**A, B**) The expression of Atrogin-1 and MuRF-1 (muscle atrophy-related markers) and MyoD (differentiation marker) in C2C12 myotubes were measured via RT-qPCR. The mRNA expression level was expressed as a ratio after normalization with the levels of β-actin. (**C**) Representative images of western blots for MuRF-1 protein extracted from C2C12 myotubes. The MuRF-1 band can be observed at the 40-kDa marker position. The relative ratio of protein/β-actin protein is represented as the fold change of the DIF control group (**D**). Data are expressed as the mean ± SD. *p < 0.01 vs. DIF control, #*p* < 0.01 vs. DIF + DEX control.

The ubiquitin-proteasome system (UPS) is a proteolytic system involved in skeletal muscle atrophy [45]. Skeletal muscle protein degradation is regulated by muscle-specific E3 ubiquitin ligases, such as Muscle RING finger 1 (MuRF1) and muscle atrophy F-Box (atrogin-1/MAFbx) [46]. MuRF1 and atrogin-1 inhibit the PI3K-Akt signaling pathway and protein synthesis by downregulating mTOR expression [30]. In particular, MuRF1 is involved in the degradation of muscle fiber proteins, such as actin and MHC, and atrogin-1 plays a role in regulating protein synthesis [47]. Expression of both ubiquitin ligases has been shown to be markedly increased in skeletal muscle atrophy [48]. Furthermore, the expression of MuRF1 and atrogin-1 is known to increase with DEX treatment [49]. Thus, RT-qPCR was performed to confirm atrogin-1 and MuRF-1 mRNA expression. Compared with those in the DIF group, atrogin-1 and MuRF1 mRNA levels were increased in the control group treated with DEX alone, confirming that DEX increased the expression of muscle-specific E3 ligases. By contrast, MuRF1 and atrogin-1 mRNA levels, which were upregulated by DEX treatment, were significantly decreased following treatment with 1% or 2% CM (Fig. 4A). Similarly, MuRF1 protein levels were increased following treatment with DEX but significantly lowered upon treatment with 1% or 2% CM (Figs. 4C and 4D). Myogenic differentiation factor D (MYOD) is a muscle-specific nuclear transcription factor that regulates the proliferation and differentiation of myoblasts and is degraded by the UPS or by autophagy [50, 51]. MYOD acts as a transcriptional activator of muscle differentiation and myogenic determination [52, 53]. MYOD levels, a muscle differentiation marker, were significantly decreased following DEX treatment compared with those in the DIF control group (Fig. 4B). Further, MYOD expression, which was decreased by DEX treatment, was significantly increased by treatment with CM.

These results indicated that the CM of *L. rhamnosus* JY02 inhibited muscle protein degradation and promoted differentiation. Therefore, the results of the in vitro experiments in which metabolites derived from *L. rhamnosus* JY02 alleviated DEX-induced muscle atrophy suggest the possibility of its use as an agent that ameliorates muscle atrophy.

### *L. rhamnosus* JY02 Alleviates DEX-Induced Muscle Atrophy in Mice.

**Fig. 5:**
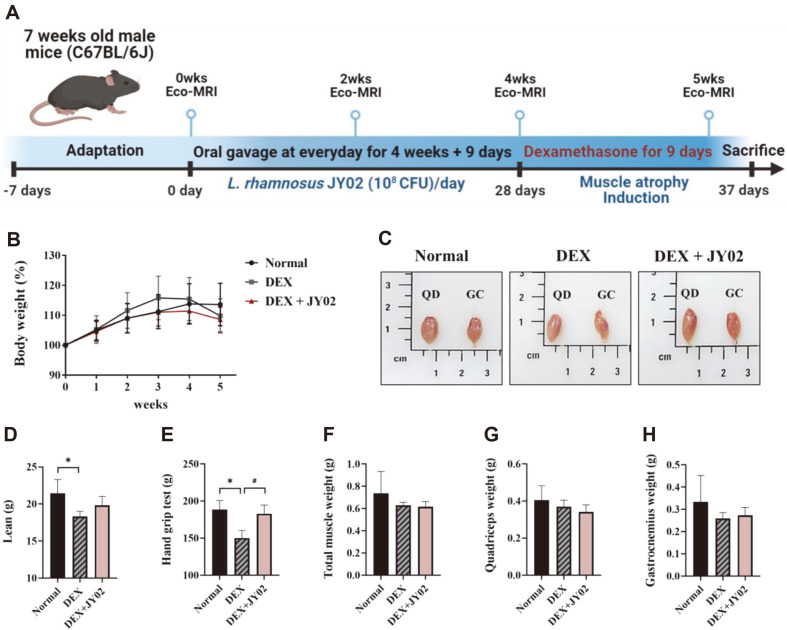
Effects of *L. rhamnosus* JY02 on dexamethasone-induced muscle atrophy in mice. (**A**) Schematic of experimental schedules. (**B**) Body weight changes in each group. (**C**) Pictures of the quadriceps (QD) and gastrocnemius (GC) muscles in each group. (**D**) Lean body mass (**E**) Grip strength measurement for muscle function evaluation. (**F**) The weight of the total muscle tissue (quadriceps and gastrocnemius muscle). (**H**) Measurement of quadriceps and gastrocnemius muscle mass. Data are expressed as the mean ± SD. *p < 0.01 vs. Normal control, #*p* < 0.01 vs. DEX control.

To evaluate whether the anti-atrophic efficacy confirmed in vitro is also effective in a mouse model, we administered live cells to a DEX-induced mouse model and evaluated its effectiveness in alleviating muscle atrophy. We used a mouse model of DEX-induced muscle loss because high-dose and long-term DEX exposure leads to decreased protein synthesis and increased protein degradation, resulting in muscle atrophy [27, 54]. Several studies have documented that supplementing *Lactobacillus* spp. At 10^8^ CFU/day/mouse modulates gut microbiota composition and enhances muscle function [55, 56]. Therefore, we chose the dose of JY02 based on the other reports. The mice were pretreated by oral gavage of JY02 suspension (10^8^ CFU/day/mouse) for four weeks. Muscle atrophy was induced via the intraperitoneal injection of dexamethasone (DEX) (20 mg/kg, once/day) for nine days (Fig. 5A). To confirm whether muscle atrophy was induced and evaluate the efficacy of the amelioration of atrophy, we measured the levels of various parameters of muscle atrophy, such as body weight, lean body mass, grip strength, and weight of muscle tissues (gastrocnemius and quadriceps femoris).

At four weeks, the body weight tended to increase; the DEX-treated group showed weight loss after treatment with DEX (Fig. 5B). Dexamethasone (DEX) induces muscle degeneration and weight loss in young mice [3]. Furthermore, it can be expected that significant changes in DEX-induced protein turnover can occur during weight loss. Body mass comprises body fat and lean body mass, including the mass of muscles, organs, and bones [57]. Because organ and bone weights do not change significantly, a decrease in lean mass suggests a reduction in skeletal muscle mass [58]. The lean mass showed a significant decline in the DEX group (18.33 ± 0.68) compared to the normal group (21.43 ± 1.88), and it was confirmed that the value slightly increased after treatment with JY02 (19.82 ± 1.21) (Fig. 5D). These results implied that the DEX-induced decrease in lean mass was slightly mitigated by the consumption of JY02. DEX treatment reduces grip strength in mice [59]. Grip strength was measured to confirm changes in muscle strength and function in mice [60]. The mice from the DEX-treated group (150.18 ± 10.08) had significantly lower grip strength than those from the normal group (188.6 ± 12.10). It was confirmed that the DEX-induced weakening of grip strength was considerably reversed by JY02 treatment (182.88 ± 11.69)(Fig. 5E). These results indicate that muscle strength can be improved by JY02 treatment. Leg muscle weights were compared to evaluate whether there was a decrease in muscle mass due to DEX exposure. The quadriceps and gastrocnemius muscles, which represent the leg muscles, were examined. There was no significant difference between the weights of the gastrocnemius and quadriceps muscles in the mice from each group, but the average weight of the gastrocnemius or quadriceps muscle in the DEX-treated mice was lower than that of the corresponding muscle from normal mice (Figs. 5C and 5F–5H–5H). As there was no significant difference between the weight of the leg muscles of the mice from the groups, a more detailed analysis was performed to confirm the muscle fiber cross-sectional area.

**Fig. 6:**
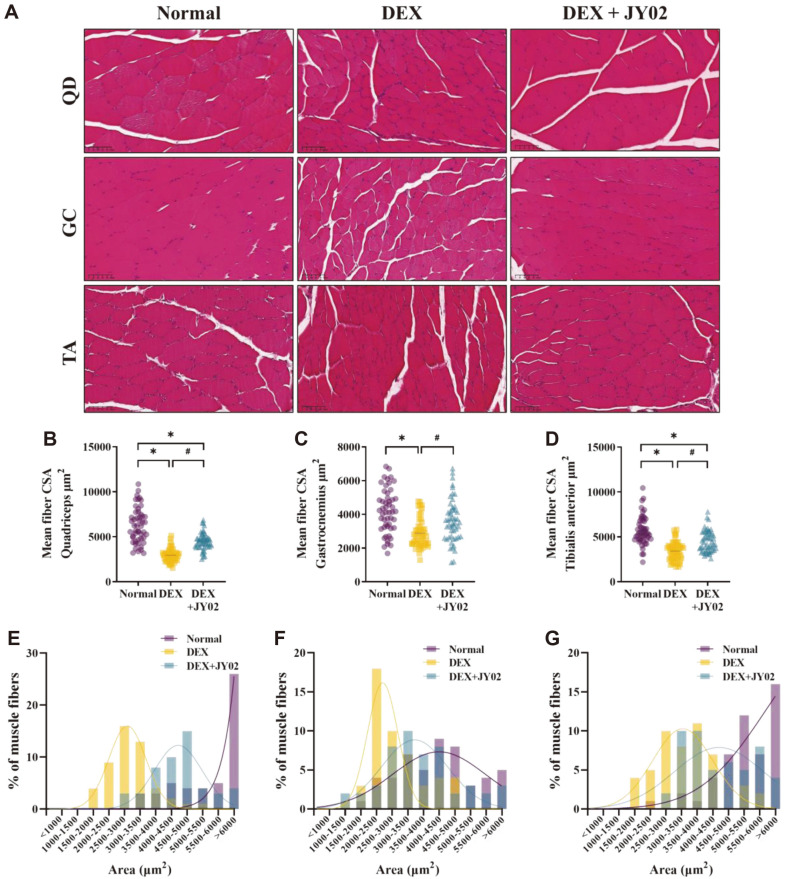
Effect of *L. rhamnosus* JY02 on the muscle fiber cross-sectional area. (**A**) Effects of JY02 on muscle atrophy (QD, GC, and TA) after DEX treatment. The muscle tissues were observed at 200× by H&E staining, and the fiber crosssectional area (μm^2^) of the muscle tissue was measured using Image J. (**B–D**) Quantification of muscle fibers CSA. (**E–G**) Distribution of the muscle fibers according to CSA ranges: 1000–6000 μm^2^. Data are expressed as the mean ± SD. *p < 0.01 vs. Normal control, #*p* < 0.01 vs. DEX control.

To accurately confirm the changes in muscular atrophy, the cross-sectional areas of the muscle fibers were compared using H&E staining. Long-term DEX treatment inhibits protein synthesis in the leg muscles, reducing the cross-sectional area of t he muscle [61]. H&E staining was performed on transverse paraffin sections of skeletal muscle to evaluate the effects of JY02 on the cross-sectional area of myofibers showing DEX-induced atrophy (Fig. 6A). Treatment of mice with 20 mg/kg DEX for 9 days significantly reduced the cross-sectional areas of the quadriceps (QD), gastrocnemius (GC), and tibialis anterior (TA) muscles. The cross-sectional area of muscle fibers in mice from the DEX group (QD: 2932.51 cm^2^, GC: 2878.85 cm^2^, and TA: 3419.47 cm^2^) was significantly less than that of the muscle fibers in mice from the normal group (QD: 6287.22 cm^2^, GC: 4211.75 cm^2^, and TA: 5735.63 cm^2^) (Figs. 6B–6D). Conversely, the cross-sectional area of muscle fibers in mice from the JY02 group increased significantly (QD: 4516.19 cm^2^, GC: 3639.64 cm^2^, and TA: 4529.63 cm^2^) compared with that of muscle fibers in mice from the DEX group. In addition, compared to the muscle fiber size distribution in mice from the DEX group, the muscle fiber size distribution in the mice from the JY02 group shifted to the right, indicating a larger cross-sectional area (Figs. 6E–6G). This suggests that the cross-sectional area of s keletal muscle in mice was increased by JY02 treatment. Although JY02 did not enhance the weights of the GC and QD weights, it enhanced the lean body mass and strength function and mitigated the reduction in muscle fiber cross-sectional area. This suggests that JY02 alleviates muscle atrophy and reduces the muscle function.

### *L. rhamnosus* JY02 Has Anti-Atrophy and Anti-Inflammatory Effects in DEX-Induced Mice

**Fig. 7:**
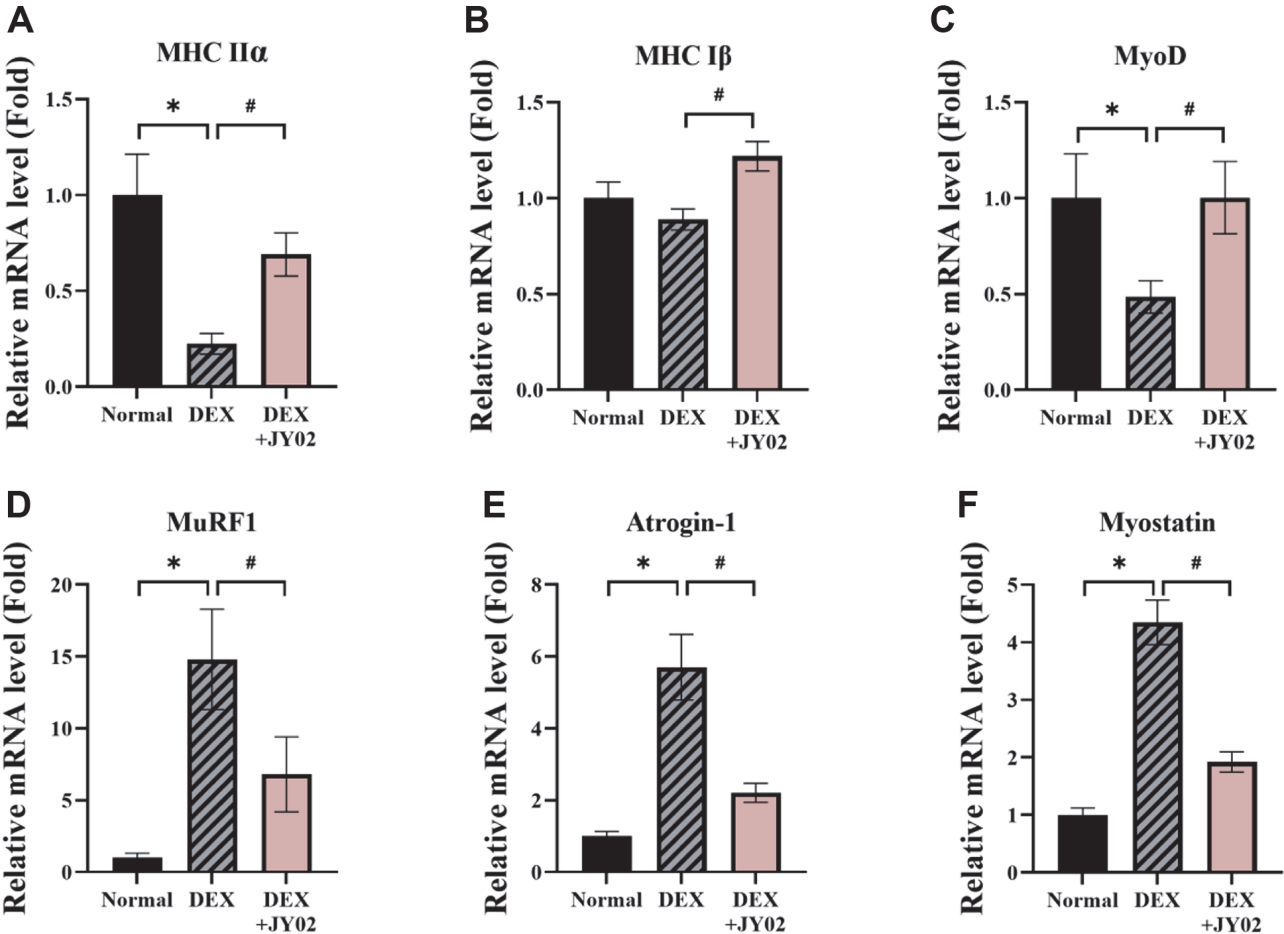
Effect of *L. rhamnosus* JY02 on mRNA and protein expression levels of muscle markers in mice tissues. (**A–F**) mRNA expression levels of muscle-related markers in atrophied quadriceps muscle after DEX treatment. Expression of muscle contraction-related (MHCIIα and MHCI β), muscle differentiation-related (MyoD), and muscle degradation-related markers (MuRF-1, Atrogin-1, and Myostatin). The levels of proteins (MuRF1 and Atrogin-1) in serum were assessed using the CBA kit. Data are expressed as the mean ± SD. *p < 0.01 vs. Normal control, #*p* < 0.01 vs. DEX control.

**Fig. 8:**
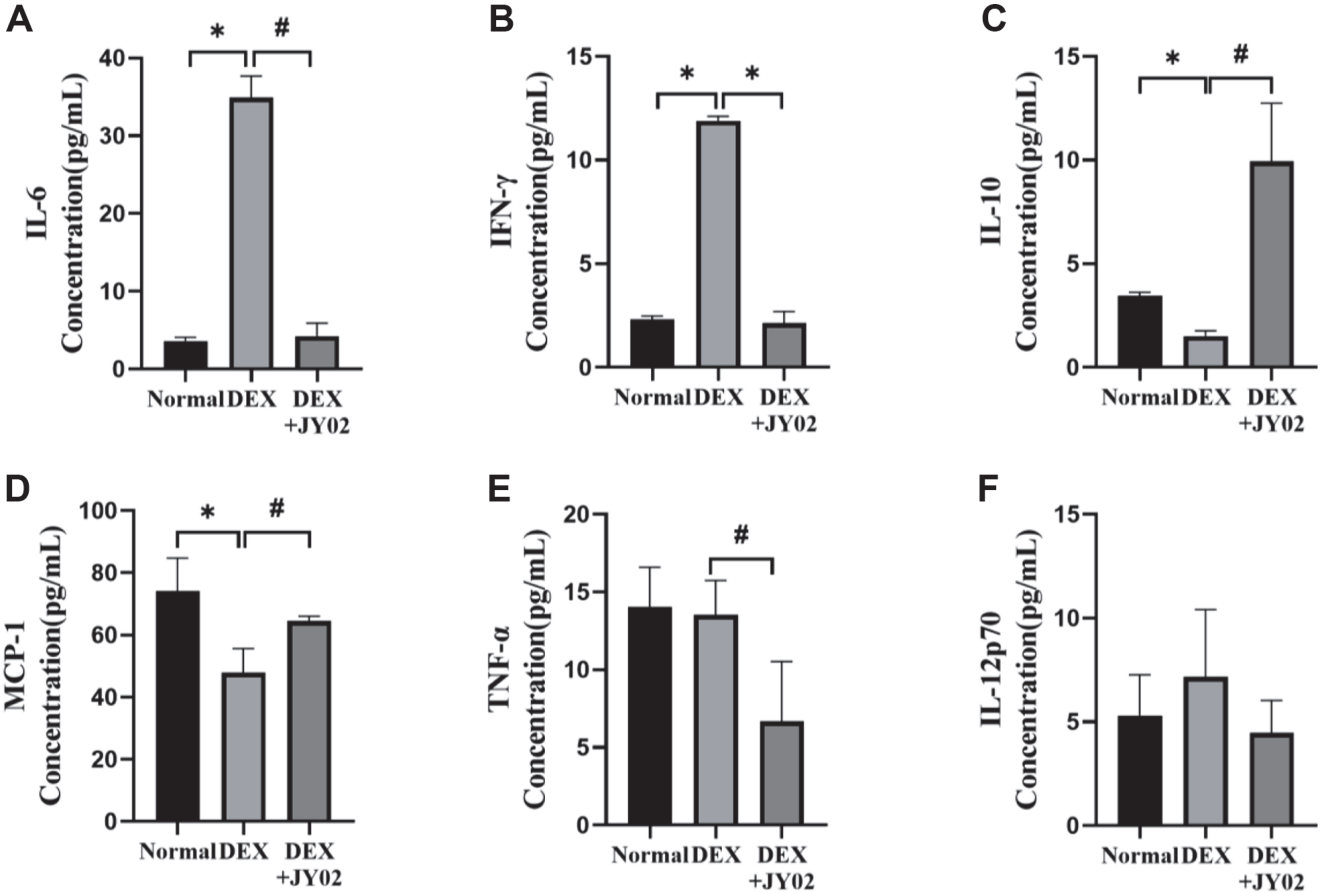
Effects of JY02 on expression of cytokines in DEX-induced sarcopenic mice. Serum levels of the proinflammatory cytokines (**A**) IL-6, (**B**) IFN-γ, (**C**) anti-inflammatory cytokines IL-10, (**D**) chemokine levels of MCP-1, (**E**) TNF-α, and (**F**) IL-12p70. Statistical differences among the groups were analyzed using one-way ANOVA and Tukey’s multiple comparison test. The results are mean ± SD of five independent experiments, *p < 0.01 vs. normal, #*p* < 0.01 vs. only DEX group.

Myosin heavy chain (MHC) is a motor protein in skeletal muscles; it produces MHC isoforms with different expression patterns. Each isoform possesses properties that define different types of muscles [62]. It has been reported that DEX treatment induced the transition of MHC isoforms in fast-to-slow type II glycolytic muscle fibers in skeletal muscles [63]. In accordance with this, DEX treatment induces a decrease in the levels of the fast-type muscles in DEX-treated muscle tissue [63, 64]. The MHCs MHCIIα and MHCIβ showed higher expression in the mice treated with JY02 than in those treated with DEX alone (Figs. 7A and 7B). In addition, the expression of MyoD, which promotes muscle differentiation, recovered significantly after treatment with JY02 (Fig. 7C). These results suggest that *L. rhamnosus* JY02 inhibits muscle atrophy by inhibiting muscle breakdown and promoting the release of MHC. In addition, it was confirmed that the levels of the ubiquitin E3 ligases MuRF-1 and atrogin-1, which were overexpressed after DEX treatment, and myostatin, which inhibits muscle breakdown marker muscle formation, were significantly lowered by treatment with JY02 (Figs. 7D–7F). Myostatin is one of the major negative regulators of muscle function; it inhibits myogenesis [65]. Myostatin affects skeletal muscle generation and growth by inhibiting Akt/mammalian target of rapamycin (mTOR) signaling and protein synthesis [66]. As DEX inhibits the function of satellite cells, DEX treatment results in the upregulation of myostatin [67]. We assessed the expression levels of five cytokines (IL-6, IFN-γ, IL-10, TNF-α, and IL-12p70) and chemokines of MCP-1 in mouse serum to determine the anti-inflammatory effects of JY02 on DEX-induced muscle atrophy. As shown in Fig. 8, JY02 pretreatment decreased pro-inflammatory factors levels (IL-6 and IFN- γ) and enhanced levels of IL-10 compared with the DEX-treated group. However, there was no significant difference in serum levels of two cytokines (TNF-α, and IL-12p70) between the normal and DEX-treated groups (Figs. 8E and 8F). Sarcopenia was associated with imbalance of cytokines and especially increased levels of inflammatory cytokine IL-6 [68]. Human studies have proposed that older adults with sarcopenia exhibit impaired “inflammatory states” characterized by higher IL-6 and IL-10 concentrations and IL-6/IL-10 ratios [69, 70]. However, the DEX-induced muscle disease model showed a decrease in IL-10 compared to the control group, which was significantly increased when mice were fed with JY02. Pro- and anti-inflammatory cytokine markers through studies using aging animal models are considered to be quite consistent with the results of human studies, and the immunomodulatory function of probiotics will be explained to improve sarcopenia. In this study, we presented biological evidence for the improvement of muscle atrophy by *L. rhamnosus* JY02 strains isolated from kimchi, and future aging mouse models will explain more about the microbiome and immune regulatory mechanisms.

## Conclusion

In this study, the kimchi-derived strain L.rhamnosus JY02 reduced atrophic muscle factors and pro-inflammatory cytokines and generated muscle differentiation markers, showing both anti-atrophy and anti-inflammation attributes. *L. rhamnosus* JY02-conditioned medium (CM) inhibited DEX-induced myotube and myofiber atrophy and expression of muscle degradation markers (MuRF1 and atrogin-1) in C2C12 cells. In addition, *L. rhamnosus* JY02 supplementation generated muscle-enhancement markers (MHC Iβ, MHC Iiα, and Myo-D) and decreased muscle atrophy-related markers, pro-inflammatory cytokines, and symptoms in mice. Overall, these results suggest that *L. rhamnosus* JY02 could be used as an anti-atrophy probiotics supplement to improve muscle function and quality.

## Supplemental Materials

Supplementary data for this paper are available on-line only at http://jmb.or.kr.
