# Supplementary material for: Lactobacillus rhamnosus JY02 Ameliorates Sarcopenia by Anti-Atrophic Effects in a Dexamethasone-Induced Cellular and Murine Model
Source: J Microbiol Biotechnol. 2023 Mar 27;33(7):915–25. doi: 10.4014/jmb.2303.03001 (PMC10394339; doi:10.4014/jmb.2303.03001)
Supplement: Supplementary file 1 [file jmb-33-7-915-supple.pdf]

J. Microbiol. Biotechnol.
https://doi.org/10.4014/jmb.2301.03001

Supplementary Information

Materials and Methods

Isolation of Lactic Acid Bacteria from Kimchi

12 types of kimchi were collected and samples were ten-fold diluted on 0.1% peptone water. The supernatant was spread on a pH 5 MRS agar plate, and then cultured at 37℃ for 2 days to identify and isolate dominant bacteria growing at high dilution rates. After incubation, ten colonies with different morphologies were randomly picked from each pH 5 MRS agar plate. All isolates were preliminarily identified as LAB depending on morphological and biochemical characteristics.

Acid tolerance, Bile acid Tolerance, and Intestinal Adhesion Capacity

To reflect the acidic conditions of the stomach, MRS broth was adjusted to pH 2.5 using 6N HCl. After autoclaving, porcine gastric mucosa-derived pepsin (Sigma- Aldrich, USA) was added to a final concentration of 1,000 units/mL and sterilized using a membrane syringe filter having a pore size of 0.45 µm. To evaluate the acid resistance of the strain, 100 µL (1%) of the strain culture was inoculated into 10 mL of an acidic solution and incubated at 37℃ for 2h. The culture media was spread on an MRS agar plate to measure the number of viable cells, and the number of viable cells incubated for 0 h was compared with the 2 hours incubated viable cells as a control.

To form an artificial bile acid conditioned medium, 0.5% oxgall was added to MRS Broth and used after autoclaving. 100 µL (1%) of lactic acid bacteria inoculated into 10ml of MRS broth added 0.5% oxgall and incubated for 24 h. After transferring the culture medium to a 96-well plate, absorbance was measured at the wavelength of 600 nm. Bile acid resistance was expressed as a growth percentage by comparing the absorbance at 0 h and at 24 h.

HT-29 cells were cultured in RPMI (Hyclone) containing 10% fetal bovine serum (FBS-Heat-Inactivated; #S 101-07, welgene), 1% Non-Essential amino acid (MEM NEAA; Gibco) and 1% Anti-anti (Antibiotic; Gibco) in a 37℃ with 5% CO_2 incubator. to confirm the ability to adhere to HT-29 cells, HT-29 cells were seeded into a 24-well plate at a concentration of $4×10^5$ /cm^2 and used when confluency reached 90%. 500 μL of 10X concentrated lactic acid bacteria were resuspended into 500 μL of pure RPMI medium (without 1% antibiotic) and added in each well and plates were incubated for 2 h at 37℃, 5% CO_2 conditions. After incubation, plates were washed three times with PBS while stirring at a speed of 200 rpm for 3 minutes each to remove non-adhered lactic acid bacteria. Cells isolated by trypsinization were diluted with 0.1% peptone water, spread on MRS Agar plates, incubated at 37℃ for 48 hours, and viable cells were counted.

Measurement of GFP, Life Span Assay, and Movement Assay Using *C. elegans*

C. elegans were synchronized and placed onto Nematode Growth Medium (NGM) Agar plates seeded with concentrated *Escherichia coli* OP50 and incubated to the L4 stage at 25℃.

For the fluorescence expression experiment, AY102*[vha-6p::pmk-1::GFP* + *rol-6(su1006)]* species was used to confirm the expression of *pmk-1*::GFP and PD4251, *myo-3*::GFP transgene expressing GFP in muscle, was used. L4 state of AY102 or PD4251 was exposed to the experimental strains for 24 h or 7 days, and 5 animals were transferred to BHI Agar. After flattening a single bubble (50uL) of 2% agarose on a slide glass, 3 worms were randomly transferred, anesthetized with sodium azide (NaN_3), and observed under a fluorescence microscope (IX53, Olympus, Japan).

For life span assay, CF512 [*fer-15(b26);fem-1(hc17*)] mutants and PD4251 (*ccIs4251* and *myo-3*::GFP) mutants were used each experiments. The L4 state of *C. elegans* were transferred one by one using a platinum wire to the NGM plates about 30 animals. The

viability was determined by transferring the worms one by one to a new plate until all worms were dead and counting under a microscope.

For movement assay, PD4251 in the L4 state was exposed to the experimental strain and then transferred to a fresh plate. On the 5th and 8th days after exposure, the movements of 3 randomly selected worms were recorded for 20 seconds. A score of 3 points was given for S-shaped movements in which both the pharynx and tail moved, 2 points for motility in which only the front part of the body moved, and 1 point for motility in which only the pharynx moved without movement.

Results

Evaluation of Potential Probiotics Properties of Kimchi-Derived LAB

According to morphological and biochemical characterization, 246 strains showing Gram-positive, catalase, and KOH-negative were finally isolated (**Table S1**). Also, JY02 was included in lactic acid bacteria isolated from Mustard leaf kimchi.

To investigate the probiotics characteristics of the isolated lactic acid bacteria, acid tolerance, bile acid tolerance, and intestinal adhesion experiments were conducted. Also, *Lactobacillus rhamnosus* GG (LGG), well known as a probiotic strain, was used as a positive control. As a result of measuring the acid tolerance and bile acid tolerance of lactic acid bacteria under acidic (pH 2.5) and bile acid (0.5% oxgall) conditions, JY02 (Named as LFR20-030 in the graph) showed an excellent survival rate of more than 80% against acid resistance **(Fig. S1A)** and showed higher bile resistance than positive control LGG **(Fig. S1B)**. As a result of evaluating the adhesion ability of the strain, JY02 showed 100% adhesion rate similar to that of LGG **(Fig. S1C)**. It was confirmed that JY02 has a probiotic effect through the evaluation of acid tolerance, bile acid tolerance, and adhesion ability to the intestine.

Functional Screening Using the *C. elegans* Model.

For functional screening, an innate immunity experiment was conducted using the AY102 mutant, in which the intensity of GFP combined with the *pmk-1* gene increases when immunity increases, and a life span assay was conducted to confirm whether there is an effect of extending lifespan through anti-aging effect. JY02 showed a high expression of *pmk-1* **(Fig. S2A)** and showed a significant life span extension effect **(Fig. S2B)**. Fluorescence expression of the *myo-3* gene was evaluated on the 7th day after exposure to the experimental bacteria using PD4251 mutant, in which GFP is conjugated to the myosin heavy chain gene that activates cytoskeletal motor activity in the body wall muscles and vulval muscles.

As a result of quantifying the expression of the *myo-3* promoter in body wall muscle as the area of muscle nucleus or the amount of GFP expression per area, JY02 showed significantly wider and higher *myo-3*::GFP expression compared to OP50 control **(Fig. S3A).** JY02 also significantly extended the life span of PD4251**(Fig. S3B)**. In a movement experiment to evaluate whether it is effective in improving the movement that decreases over time, it was confirmed that the movement of JY02 remained high on the 8th day **(Fig. S3C)**. These results suggest the potential of JY02 to alleviate or delay age-related muscle decline.

JY02, which improved age-related movement, was finally selected and confirmed to belong to the genus *L. rhamnosus* through 99% (1494/1509) identical to the 16s rRNA gene of *L. rhamnosus* strain JCM1136 (accession No: NR_043408). 16s rRNA sequence from isolated JY02 was shown in **Table S2** and the phylogenetic tree shown in **Fig. S4.**

***L. rhamnosus* JY02 Modulates Levels of Pro-Inflammatory Cytokines in DEX-Induced Mice.**

We assessed the expression levels of five cytokines (IL-6, IFN-γ, IL-10, TNF-α, and IL-12p70) and chemokines of MCP-1 in mouse serum to determine the anti-inflammatory effects of JY02 on DEX-induced muscle atrophy. As shown in Fig S5, JY02 pretreatment decreased pro-inflammatory factors levels (IL-6, IFN- γ, and MCP-1) and enhanced levels of IL-10 compared with the DEX-treated group. However, there was no significant difference in serum levels of two cytokines (TNF- α, and IL-12p70) between the normal and DEX-treated groups (Fig. S5E and F).

Supplementary Figure 1. Evaluation of Acid Resistance, Bile Acid Resistance, and Intestinal Adhesion of LAB

(A) Survival rate of strains exposed for 2 hours to gastric juices containing pepsin at pH 2.5 compared to positive control LGG (100%). Survival (%) = 2 h viable cell count / 0 h viable cell count × 100%. (B) Survival rate of strains exposed for 24 h to bile acid condition containing 0.5% oxgall compared to positive control LGG (100%). Survival (%) = 24 h viable cell count / 0 h viable cell count × 100%. (C) The adhesion of each strain was normalized by positive control LGG (100%). Survival (%) = 2 h viable cell count / 0 h viable cell count × 100%. The Results are given as mean values ± standard deviation (SD) for replicates from a single sample. * $p < 0.01$ vs. the control group (LGG).

Supplementary Figure 2. *pmk-1*::GFP and Lifespan Analysis of *C. elegans* Exposed to Probiotic Lactic Acid Bacteria.

(A) Analysis of *pmk-1*::GFP fluorescence intensity in AY102 [*vha-6p::pmk-1::GFP* + *rol-6(su1006)*] using the fluorescence microscopy (IX53, Olympus, Japan). The intensity was measured using ImageJ and divided by the number of worms. *E. coli* OP50 was used as a negative control and LGG as a positive control. (B) L4 state of CF512 [*fer-15(b26);fem-1(hc17)*] were exposed to lactic acid bacteria until death, the number of deaths by day is counted. Statistics were calculated relative to worms exposed to *E. coli* OP50. Data were performed in 3 repetitions (n = 30 per plate). * $p < 0.01$ vs. the control group (OP50).

Supplementary Figure 3. *myo-3*::GFP, Lifespan, and Movement Analysis of *C. elegans* Exposed to Probiotic Lactic Acid Bacteria.

(A) Analysis of *myo-3*::GFP fluorescence intensity in PD4251. Fluorescence expression was

measured on the 7th day after exposing worms in the L4 state to lactic acid bacteria. Photographs observed by fluorescence microscopy (IX53, Olympus, Japan). The intensity was measured using the ImageJ program. (B) L4 state of PD4251 was exposed to lactic acid bacteria until death, the number of deaths by day is counted. Statistics were calculated relative to worms exposed to *E. coli* OP50. Data were performed in 3 repetitions (n = 30 per plate). (C) Motion score of *C. elegans* on day 5 and day 8 of exposure to probiotics. * $p < 0.01$ vs. the control group (OP50).

Supplementary Figure 4. The phylogenetic position of JY02.

Phylogenic neighbor-joining JY02 by 16S rRNA sequence. JY02 is named as LFR20-030 in the graph.

Table S1. Potentially probiotic lactic acid bacteria strains isolated from Kimchi.

No.	Sources	Selected strains
1	Onion kimchi	14
2	Ginseng kimchi	26
3	Young radish water Kimchi	25
4	Coriander Kimchi	22
5	Radish kimchi	10
6	Cucumber kimchi	30
7	Mustard leaf kimchi	18
8	Green Onion kimchi	18
9	Fresh cabbage kimchi (Geotjeori)	12
10	Aged kimchi	11
11	1-year-old kimchi	37
12	2-year-old kimchi	23
Total		246

Table S2. Nucleotide sequences of 16S rRNA of JY02.

CAGATGAACGCTGGCGGCGTGCCTAATACATGCAAGTCGAACGAGTTCTGATTATTGAAAGGTG
CTTGCATCTTGATTTAATTTTGAACGAGTGGCGGACGGGTGAGTAACACGTGGGTAACCTGCCCTTA
AGTGGGGGATAACATTTGGAAACAGATGCTAATACCGCATAAATCCAAGAACCGCATGGTTCTTGGC
TGAAAGATGGCGTAAGCTATCGCTTTTGGATGGACCCGCGGCGTATTAGCTAGTTGGTGAGGTAACG
GCTCACCAAGGCAATGATACGTAGCCGAACTGAGAGGTTGATCGGCCACATTGGGACTGAGACACG
GCCCAAACTCCTACGGGAGGCAGCAGTAGGGAATCTTCCACAATGGACGCAAGTCTGATGGAGCAA
CGCCGCGTGAGTGAAGAAGGCTTTCGGGTCGTAAAACTCTGTTGTTGGAGAAGAATGGTCGGCAGA
GTAACTGTTGTCGGCGTGACGGTATCCAACCAGAAAGCCACGGCTAACTACGTGCCAGCAGCCGCG
GTAATACGTAGGTGGCAAGCGTTATCCGGATTTATTGGGCGTAAAGCGAGCGCAGGCGGTTTTTTAA
GTCTGATGTGAAAGCCCTCGGCTTAACCGAGGAAGTGCATCGGAAACTGGGAAACTTGAGTGCAGA
AGAGGACAGTGGAACTCCATGTGTAGCGGTGAAATGCGTAGATATATGGAAGAACACCAGTGGCGA
AGGCGGCTGTCTGGTCTGTAACTGACGCTGAGGCTCGAAAGCATGGGTAGCGAACAGGATTAGATA
CCCTGGTAGTCCATGCCGTAAACGATGAATGCTAGGTGTTGGAGGGTTTCCGCCCTTCAGTGCCGCA
GCTAACGCATTAAGCATTCCGCCTGGGGAGTACGACCGCAAGGTTGAAACTCAAAGGAATTGACGG
GGGCCCGCACAAGCGGTGGAGCATGTGGTTTAATTCGAAGCAACGCGAAGAACCTTACCAGGTCTT
GACATCTTTTGATCACCTGAGAGATCAGGTTTCCCCTTCGGGGGCAAAATGACAGGTGGTGCATGGT
TGTCGTCAGCTCGTGTCGTGAGATGTTGGGTTAAGTCCCGCAACGAGCGCAACCCTTATGACTAGTT
GCCAGCATTTAGTTGGGCACTCTAGTAAGACTGCCGGTGACAAACCGGAGGAAGGTGGGGATGACG
TCAAATCATCATGCCCCTTATGACCTGGGCTACACACGTGCTACAATGGATGGTACAACGAGTTGCGA
GACCGCGAGGTCAAGCTAATCTCTTAAAGCCATTCTCAGTTCGGACTGTAGGCTGCAACTCGCCTAC
ACGAAGTCGGAATCGCTAGTAATCGCGGATCAGCACGCCGCGGTGAATACGTTCCCGGGCCTTGTAC
ACACCGCCCGTCACACCATGAGAGTTTGTAACACCCGAAGCCGGTGGCGTAACCCTTTTAGGGAGC
GAGCCGTCTAAGGTGGGACAAATGATTAGGGTGAAGTCGTACAAAGGG

Figure S1

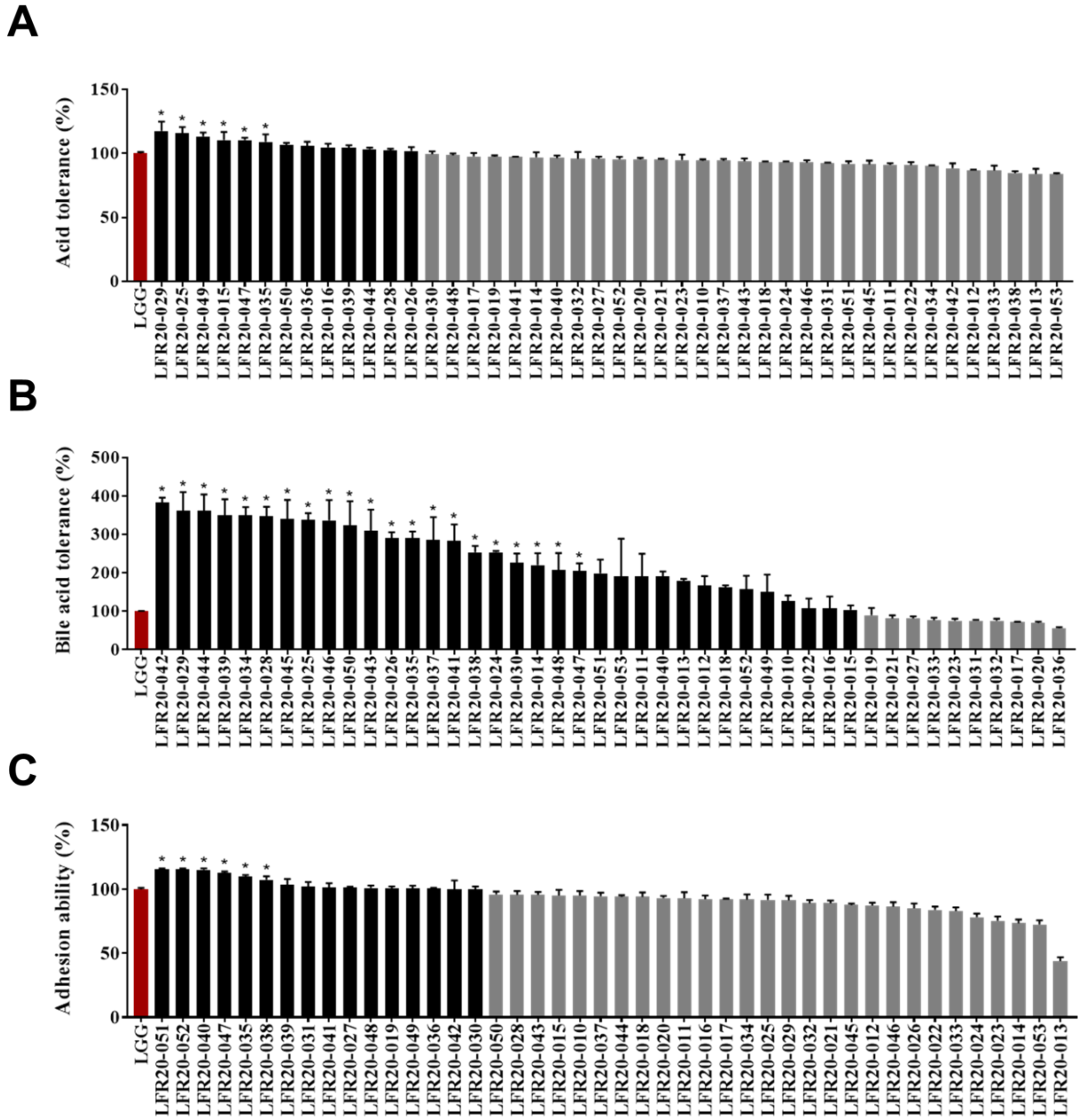
A
Acid tolerance (%)
150
100
50
0
LGG
LFR20-029
LFR20-025
LFR20-049
LFR20-015
LFR20-047
LFR20-035
LFR20-050
LFR20-036
LFR20-016
LFR20-039
LFR20-044
LFR20-028
LFR20-026
LFR20-030
LFR20-048
LFR20-017
LFR20-019
LFR20-041
LFR20-014
LFR20-040
LFR20-032
LFR20-027
LFR20-052
LFR20-020
LFR20-021
LFR20-023
LFR20-010
LFR20-037
LFR20-043
LFR20-018
LFR20-024
LFR20-046
LFR20-031
LFR20-051
LFR20-045
LFR20-011
LFR20-022
LFR20-034
LFR20-042
LFR20-012
LFR20-033
LFR20-038
LFR20-013
LFR20-053
B
Bile acid tolerance (%)
500
400
300
200
100
0
LGG
LFR20-042
LFR20-029
LFR20-044
LFR20-039
LFR20-034
LFR20-028
LFR20-045
LFR20-025
LFR20-046
LFR20-050
LFR20-043
LFR20-026
LFR20-035
LFR20-037
LFR20-041
LFR20-038
LFR20-024
LFR20-030
LFR20-014
LFR20-048
LFR20-047
LFR20-051
LFR20-053
LFR20-011
LFR20-040
LFR20-013
LFR20-012
LFR20-018
LFR20-052
LFR20-049
LFR20-010
LFR20-022
LFR20-016
LFR20-015
LFR20-019
LFR20-021
LFR20-027
LFR20-033
LFR20-023
LFR20-031
LFR20-032
LFR20-017
LFR20-020
LFR20-036
C
Adhesion ability (%)
150
100
50
0
LGG
LFR20-051
LFR20-052
LFR20-040
LFR20-047
LFR20-035
LFR20-038
LFR20-039
LFR20-031
LFR20-041
LFR20-027
LFR20-048
LFR20-019
LFR20-049
LFR20-036
LFR20-042
LFR20-030
LFR20-050
LFR20-028
LFR20-043
LFR20-015
LFR20-010
LFR20-037
LFR20-044
LFR20-018
LFR20-020
LFR20-011
LFR20-016
LFR20-017
LFR20-034
LFR20-025
LFR20-029
LFR20-032
LFR20-021
LFR20-045
LFR20-012
LFR20-046
LFR20-026
LFR20-022
LFR20-033
LFR20-024
LFR20-023
LFR20-014
LFR20-053
LFR20-013

Figure S2

A

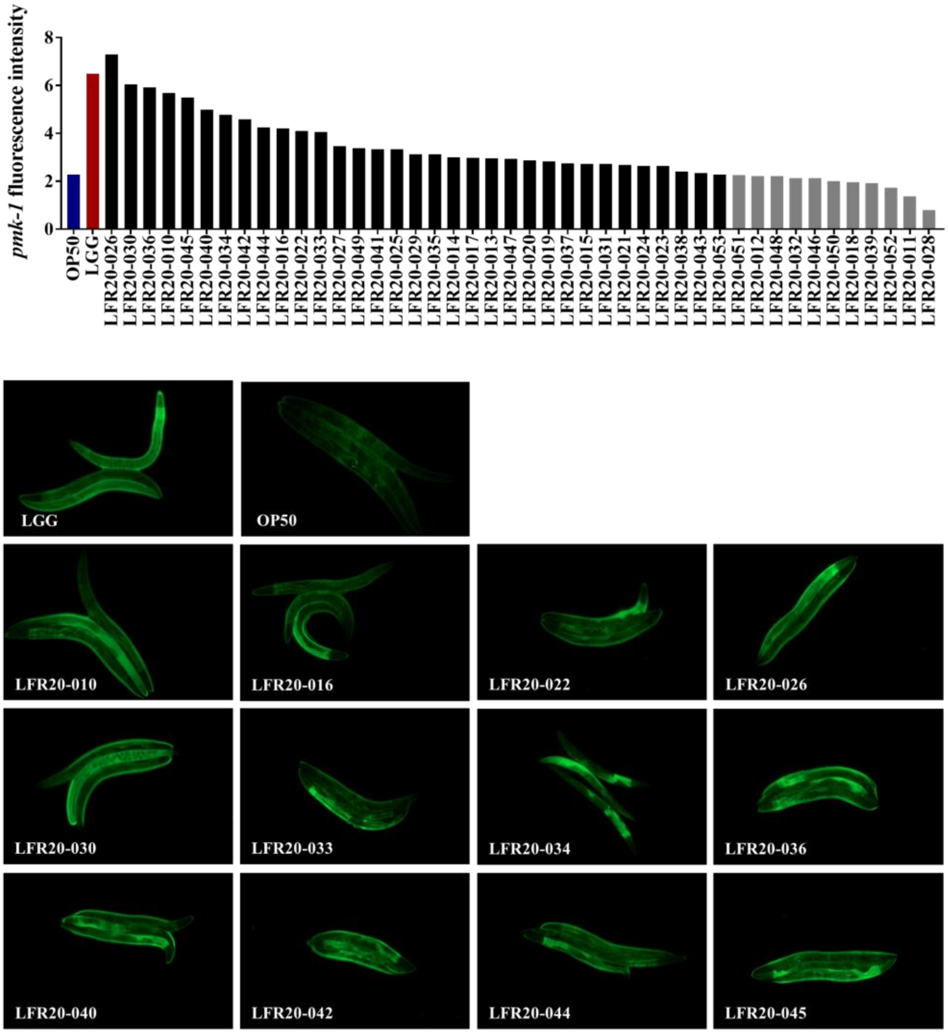

pmk-1 fluorescence intensity
0
2
4
6
8
OP50
LGG
LFR20-026
LFR20-030
LFR20-036
LFR20-010
LFR20-045
LFR20-040
LFR20-034
LFR20-042
LFR20-044
LFR20-016
LFR20-022
LFR20-033
LFR20-027
LFR20-049
LFR20-041
LFR20-025
LFR20-029
LFR20-035
LFR20-014
LFR20-017
LFR20-013
LFR20-047
LFR20-020
LFR20-019
LFR20-037
LFR20-015
LFR20-031
LFR20-021
LFR20-024
LFR20-023
LFR20-038
LFR20-043
LFR20-053
LFR20-051
LFR20-012
LFR20-048
LFR20-032
LFR20-046
LFR20-050
LFR20-018
LFR20-039
LFR20-052
LFR20-011
LFR20-028
LGG
OP50
LFR20-010
LFR20-016
LFR20-022
LFR20-026
LFR20-030
LFR20-033
LFR20-034
LFR20-036
LFR20-040
LFR20-042
LFR20-044
LFR20-045

B

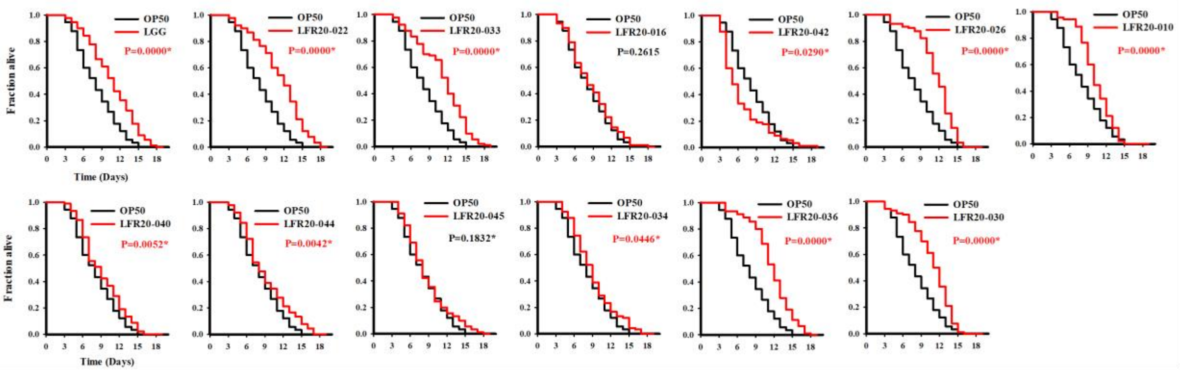

Fraction alive
Time (Days)
OP50
LGG
P=0.0000*
LFR20-022
P=0.0000*
LFR20-033
P=0.0000*
LFR20-016
P=0.2615
LFR20-042
P=0.0290*
LFR20-026
P=0.0000*
LFR20-010
P=0.0000*
LFR20-040
P=0.0052*
LFR20-044
P=0.0042*
LFR20-045
P=0.1832*
LFR20-034
P=0.0446*
LFR20-036
P=0.0000*
LFR20-030
P=0.0000*

Figure S3

A

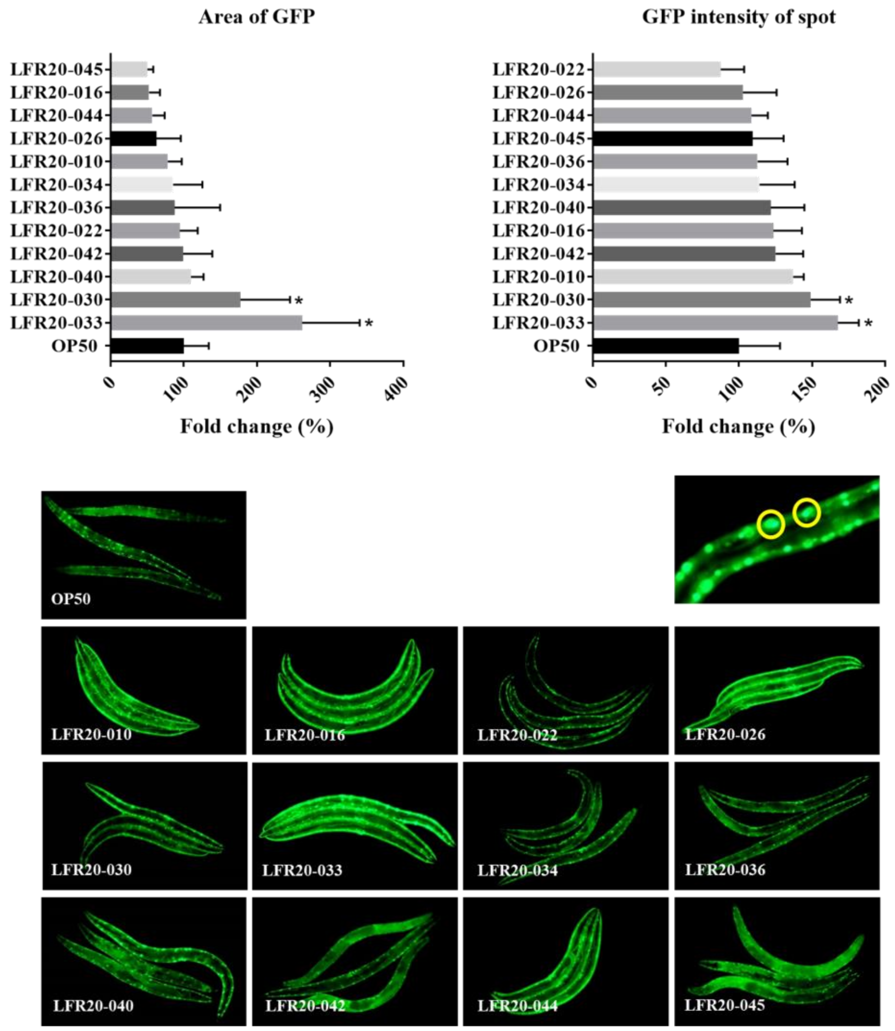

B

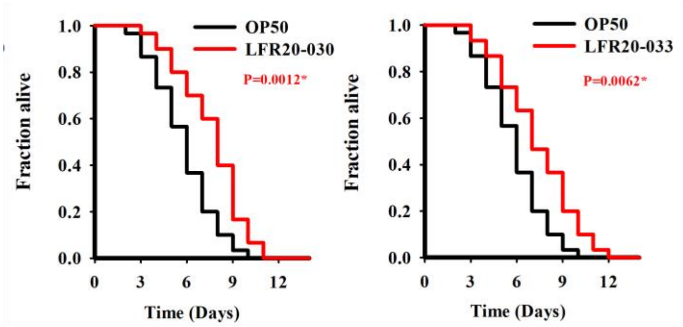

C

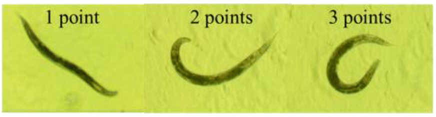

Score	Motion
1 point	There is no movement as a whole, only the pharynx moves
2 points	movement of the front part of the body
3 points	S-shaped movement

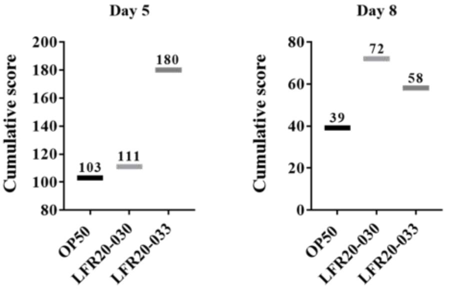

Figure S4

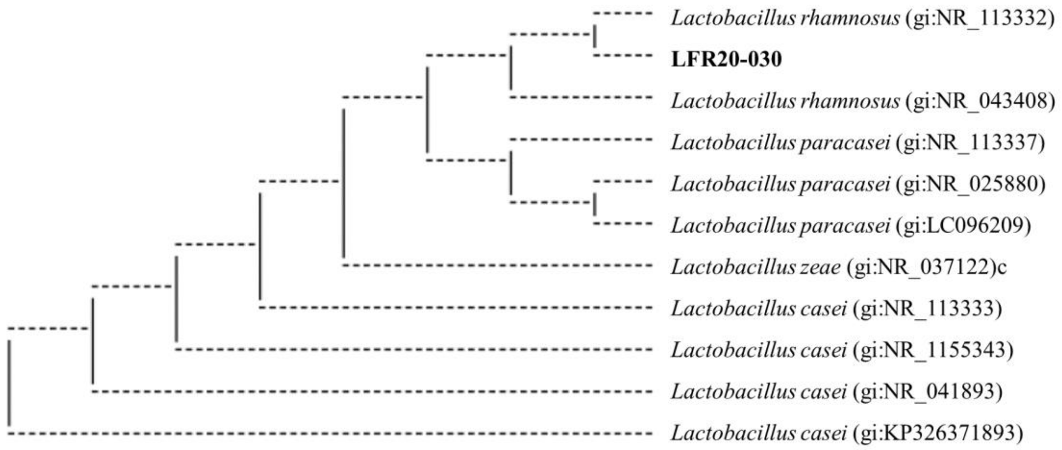

Lactobacillus rhamnosus (gi:NR_113332)
LFR20-030
Lactobacillus rhamnosus (gi:NR_043408)
Lactobacillus paracasei (gi:NR_113337)
Lactobacillus paracasei (gi:NR_025880)
Lactobacillus paracasei (gi:LC096209)
Lactobacillus zeae (gi:NR_037122)c
Lactobacillus casei (gi:NR_113333)
Lactobacillus casei (gi:NR_1155343)
Lactobacillus casei (gi:NR_041893)
Lactobacillus casei (gi:KP326371893)
